# Short-term, manualized schema-focused group therapy for patients with CBT-resistant disorders within primary care: a pilot study with a naturalistic pre-treatment and post-treatment design

**DOI:** 10.3389/fpsyg.2024.1349329

**Published:** 2024-03-26

**Authors:** Ingeborg L. Kiers, Hein A. de Haan

**Affiliations:** ^1^Department Centiv, Vincent van Gogh, Venray, Netherlands; ^2^Tactus Addiction Treatment, Enschede, Netherlands; ^3^Forensic Psychiatry Department de Boog, Ggnet Mental Health Institute, Warnsveld, Netherlands

**Keywords:** schema therapy, primary care, schema modes, CBT-resistant, short-term

## Abstract

The aim of this study was to explore the feasibility and preliminary effectiveness of a short-term, manualized schema therapy group for 77 patients with CBT-resistant mood and/or anxiety and/or personality disorders (PDs) in primary care. The primary focus was on the effects of this treatment on Early Maladaptive Schemas (EMS), schema modes, and psychological well-being. These aspects were assessed pre-and post-treatment treatment using the Young Schema Questionnaire (YSQ), the Schema Mode Inventory version 1.1 (SMI), and the Symptom Questionnaire-48 (SQ-48). The treatment consisted of 16 sessions, incorporating cognitive, behavioral, and experiential techniques. EMS significantly decreased from pre-treatment to post-treatment, as along with maladaptive schema modes. Adaptive modes increased, as did psychological wellbeing. There were no significant differences between the DSM-5 classifications regarding changes in the aforementioned measures, except for the maladaptive modes, where the value of the corrected within-subject effect indicated a significant interaction. *Post hoc* comparisons were therefore conducted which showed that patients with a mood disorder experienced more positive changes in maladaptive modes compared to patients with anxiety disorders and PDs (*p* < 0.001). There was no significant difference between those with PDs and those with Anxiety Disorders. Our findings provide preliminary evidence that short-term, manualized schema therapy might be an effective treatment for patients with CBT-resistant mood and/or anxiety and/or PDs in primary care.

## Introduction

Approximately 35% of individuals receiving mental healthcare in the Netherlands are treated within primary care settings (de Ruiter et al., 2017). Among this population, 25% are diagnosed with a personality disorder, 35% with a (chronic) depressive disorder, and an additional 25% with a (chronic) anxiety disorder (Roca et al., 2009; Beckwith et al., 2014; Magnée et al., 2014).

A significant proportion of patients with the aforementioned disorders continue to experience symptoms or relapses after undergoing cognitive-behavioral therapy (CBT), which is the recommended treatment for mood and anxiety disorders (Schene, 2007). This, in turn, can lead to disorders following a chronic course (Durham et al., 2003; Fournier et al., 2009; Loerinc et al., 2015; Springer et al., 2018). Research indicates that patients with persistent symptoms or relapses after receiving CBT often exhibit elevated levels of early maladaptive schemas (EMS) (Hawke and Provencher, 2011).

The term EMS, coined by Young et al. (2003), refers to “a broad, pervasive theme or pattern comprised of memories, emotions, cognitions, and bodily sensations, regarding oneself and one’s relationships with others, developed during childhood or adolescence, elaborated throughout one’s lifetime, and dysfunctional to a significant degree.” Schema therapy (ST) has emerged as a potentially effective treatment approach for addressing these maladaptive schemas in CBT-resistant patients with chronic mood and anxiety disorders, leading to symptom reduction (Hawke and Provencher, 2011; Carter et al., 2013).

It underlines the identification and mitigation of maladaptive coping behaviors, commonly known as coping modes, which are intricately linked to EMS (Young et al., 2003). These maladaptive coping strategies impede the potential for change and the alleviation of symptoms. Consequently, ST prioritizes the cultivation of healthier and more adaptive schemas and modes while facilitating the healing of unhelpful schemas and modes (Young et al., 2003). Scientific research also suggests that additional elements, such as peer-support, adding to a sense of recognition and support; opportunities for vicarious learning, and the ability to adequately express ones needs to others, are of added value in group therapy, and therefor also in group based ST (Jacob and Arntz, 2013; Renner et al., 2013; Skewes et al., 2015; Arntz et al., 2022).

Due to its prolonged duration, traditional Schema Therapy (ST) does not appear to be the most suitable option for primary care settings. However, the development of short-term group schema (cognitive-behavioral) therapy by Van Vreeswijk and Broersen (2006, 2013) has opened up new possibilities for implementing ST in primary care.

Van Vreeswijk and Broersen have devised two separate treatment protocols—one focusing on cognitive techniques (schema cognitive-behavioral therapy, or SCBT) and the other on experiential techniques (schema experiential therapy, or SEPT), which have been intended to be used either separately or together consecutively (van Vreeswijk and Broersen, 2013, 2017; Rommelse et al., 2017). Cognitive techniques center around the identification, challenging, and modification of maladaptive thoughts and beliefs. In contrast, experiential techniques concentrate on the reenactment and reliving of past experiences. Renner et al. (2013) and van Vreeswijk et al. (2012) investigated the cognitive protocol (SCBT-G). This highly structured protocol has a special emphasis on the cognitive and behavioral interventions of ST although limited reparenting and empathic confrontation are also employed. In the study by Skewes et al. the treatment was adapted from van Vreeswijk and Broersen's (2013) group schema cognitive-behavioral therapy protocol (SCBT-g; van Vreeswijk and Broersen, 2013). The adaptation was a significantly greater emphasis on schema mode work and experiential change techniques. It was a closed therapy group with provision of five individual sessions during therapy. These studies have shown that both therapies might be an effective form of treatment for patients with PDs and chronic mood and anxiety disorders (van Vreeswijk et al., 2012; Renner et al., 2013; Skewes et al., 2015). Consequently, a more condensed variant of ST, which integrates both cognitive and experiential techniques (Re-Focus), might be effective for patients in primary mental health care with PD and chronic mood and anxiety disorders, who are treatment-resistant to Cognitive Behavioral Therapy (CBT).

There have been no studies investigating the implementation of such a short-term ST that combines both cognitive and experiential approaches in primary mental health care, targeting patients with CBT-resistant personality and/or mood and/or anxiety disorders. The present paper reports findings from a pilot study involving the implementation of Re-Focus, a manualized short-term group schema therapy based on standard ST, which integrates cognitive and experiential techniques. The study was conducted within a case series comprising 77 patients diagnosed with PDs, therapy-resistant anxiety disorders, and/or mood disorders in a primary care setting. The primary objectives of this study were to investigate potential changes in EMS, modes, and overall psychological well-being. Our hypotheses posited that patients, irrespective of their DSM-5 classification, would demonstrate a reduction in general symptoms, dysfunctional schemas, and modes immediately after completing the treatment, suggesting that this intervention might be effective not only for patients with a Personality Disorder (PD) but also for individuals with other disorders. Additionally, we expected an increase in adaptive modes and overall vitality among patients. Moreover, we anticipated a low dropout rate, estimated to be around 10–15% because drop-out rates in both regular ST and short-term ST have been found to be low (Jacob and Arntz, 2013; Renner et al., 2013; Skewes et al., 2015).

## Design

The current study utilized a naturalistic single-group pre- and post-treatment design to evaluate the outcomes of Re-Focus treatment within a diverse group of participants exhibiting heterogeneous pathology, including PDs, therapy-resistant depression, and therapy-resistant anxiety disorders. These individuals sought treatment at a primary mental health care facility (Centiv). Before participating in the Re-Focus study, all patients had undergone cognitive-behavioral therapy (CBT) with either no noticeable improvement or limited effectiveness.

## Methods

### Participants

**Figure 1:**
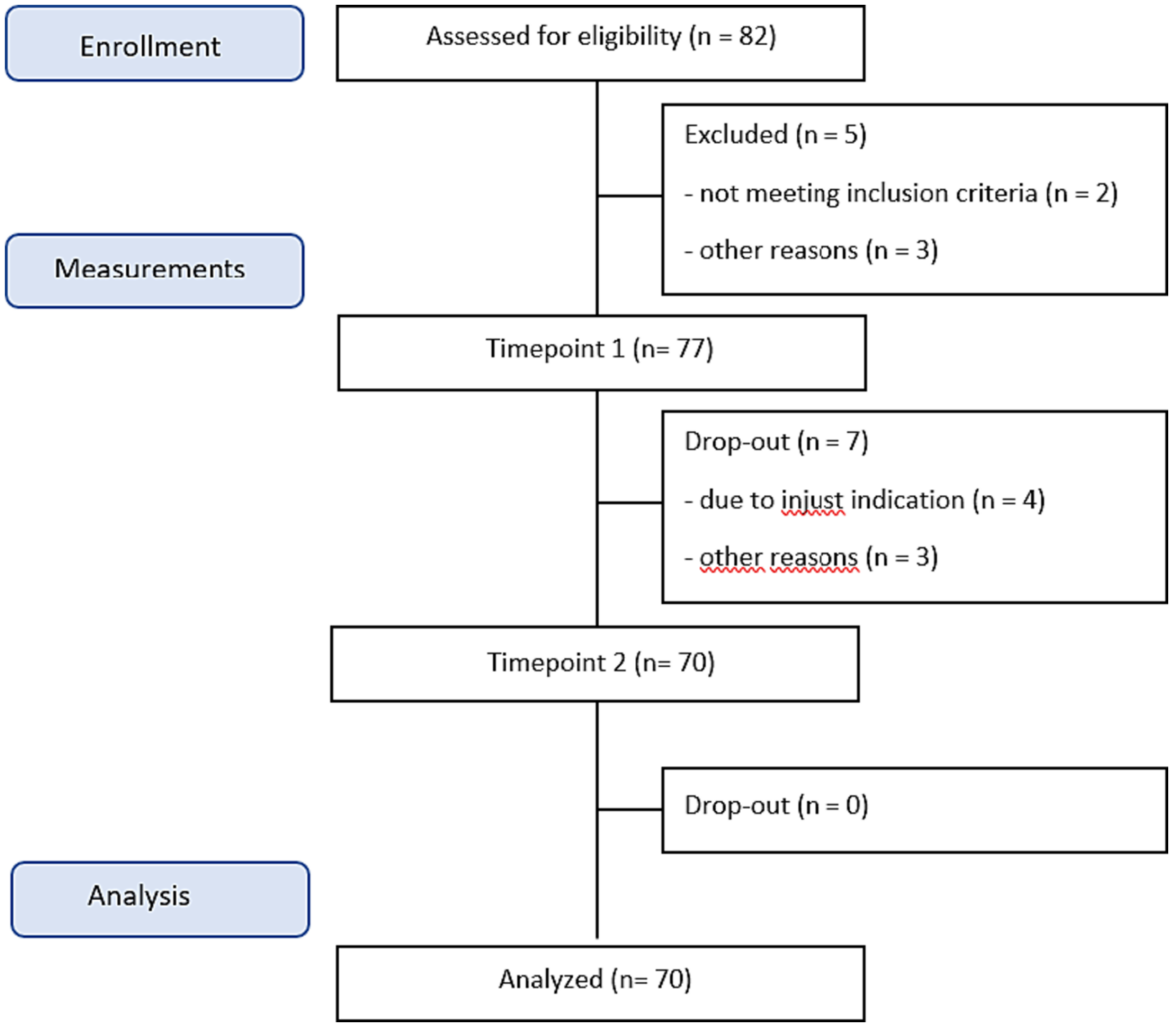
Consort diagram of the study.

Since the current study is an explorative study, we did not use a power analysis, instead we included as many participants as possible in a predetermined timeframe. A total of 77 patients were included in the period between January 2022 and June 2023 (see Figure 1). Patients were eligible for inclusion in the study if they met the following criteria: (1) aged between 18 and 65 years, (2) currently diagnosed with a personality disorder, mood disorder, or anxiety disorder according to the Diagnostic and Statistical Manual for Mental Disorders, fifth edition (DSM-5, American Psychiatric Association, 2013), and (3) had undergone at least one evidence-based treatment (CBT) for the aforementioned diagnosis in primary or secondary care. Patients were excluded from the study if they met any of the following criteria: (1) insufficient proficiency in the Dutch language, (2) a recent suicide attempt within the past three months or current active suicidal ideation, (3) diagnosed with a psychotic and/or bipolar disorder, (4) hospitalized due to self-mutilation within the past three months, (5) diagnosed with a complex dissociative disorder and/or experiencing dissociative symptoms, or (6) diagnosed with a substance use disorder, excluding tobacco, according to the DSM-5 criteria.

### Treatment

The treatment approach utilized in this study is Re-Focus, a shortened and manualized version of group schema therapy (ST) specifically designed for patients with PDs, therapy-resistant depression, and/or anxiety disorders. The first author of this paper was one of the two psychologists closely involved in developing this protocol. The development of Re-Focus draws upon the evidence-based ST treatment for personality disorders (Masley et al., 2011; Jacob and Arntz, 2013; van Vreeswijk and Broersen, 2013). The therapy is conducted in a closed group format, comprising 16 sessions and administered by two clinical psychologists trained in ST and Re-Focus. Each group accommodates a maximum of eight patients.

While incorporating all components of the original ST treatment, Re-Focus adopts a more condensed format. The manualized group therapy consists of 16 150-min weekly sessions. During the initial phase of therapy, a few sessions are dedicated to building connections and fostering a safe group climate. Participants are also educated about the schema mode model, with a focus on their top three modes. Each week, participants are asked to fill out schema mode diaries in which they also score the severity of their top three modes. Experiential and interpersonal techniques, such as limited reparenting, chair work, empathic confrontation, and imagery rescripting, are employed. These techniques aim to increase participants’ awareness of their modes and process negative experiences. This first phase comprises 6 sessions.

The second phase of the group primarily focuses on behavior change. Role plays involving healthy adult vs. parent modes, coping modes, cognitive techniques such as cost–benefit analysis and pie charts, and behavioral experiments are utilized in this phase. Participants are encouraged to integrate what they have learned in therapy into their daily lives. Therapists also promote the development of independence, autonomy, and healthy assertiveness skills for participants to recognize and meet their own emotional needs. The second phase consists of 7 sessions.

In the final phase of treatment, the focus shifts to awareness of change, finding ways to sustain these changes, and continuing the process of change. There is also time dedicated to bidding farewell to each other and the therapy. The third and last phase comprises 3 sessions (Farrell et al., 2012; Van Vreeswijk and Broersen, 2017; Reubsaet, 2018).The main difference with the protocols designed by van Vreeswijk et al. are that Re-Focus combines both experiential and cognitive techniques, it focuses more on modes than on schemas and is more divided into treatment phases, which is currently part of the standard model for individual ST. Also, the combination of both techniques is comprised into a shorter form.

Treatment integrity was ensured by adhering to the treatment manual and participating internal intervision sessions focused on the treatment protocol.

### Measures

The Young Schema Questionnaire (YSQ, Dutch version) is a 205-item self-report questionnaire assessing 15 early maladaptive schemas (Young, 1990). Each item is phrased as a negative core belief regarding oneself and one’s relations to others and is rated along a 6-point Likert scale. The YSQ has adequate to high psychometric properties, with a Cronbach’s alpha from 0.70 to 0.96 for all the 15 scales (Oei and Baranoff, 2007).

The Schema Mode Inventory version 1.1 (SMI, Dutch version) is a 118-item self-report questionnaire assessing 14 schema modes (Lobbestael et al., 2010). Each item is rated along a 6-point Likert scale. Schema modes can be divided into four categories: child modes, dysfunctional coping modes, dysfunctional parent modes, and the healthy adult mode (Young et al., 2003). Research indicates that the SMI has adequate to high psychometric properties with a Cronbach’s alpha of 0.87 for the total score and acceptable internal consistencies of the 14 subscales (Cronbach α’s from 0.79 to 0.96) (Lobbestael et al., 2010).

The Symptom Questionnaire-48 (SQ-48, Dutch version) is a 48-item self-report questionnaire assessing general psychological wellbeing (Carlier et al., 2017). Each item is rated along a 5-point Likert scale. Five subscales cover aspects of psychopathology (depression, anxiety, somatization, and agoraphobia) and four subscales cover aspects of behavior (aggression, cognitive problems, social phobia, work, and vitality/optimism). Each item is rated along a 5-point Likert scale. Research indicates that the SQ-48 has adequate to high psychometric properties with a Cronbach’s alpha ranging from 0.78 to 0.98 across the different subscales (Carlier et al., 2017).

### Procedure

Informed consent was obtained from all patients and ethical approval was granted by the Commission for Scientific Research and Participation (CSRP) of Vincent van Gogh Institute. All therapists employed at Centiv, a primary mental healthcare facility, were duly informed about the study with regards to participant recruitment during the intake phase. A screening process was conducted during intake to assess whether the patients met the inclusion criteria. Prior to participating in the study, all patients were required to provide informed consent. Treatment consisted of 16 weekly 150-min sessions. All questionnaires were administered at pre-, and post-treatment stage. These measures were an integral part of the therapy process and/or standard routine outcome monitoring (ROM). In accordance with the Young Schema Questionnaire (YSQ) and Schema Mode Inventory (SMI), patients possessed awareness of their predominant three schemas and modes that were the focal points during the initial stage of treatment. Patients retained the autonomy to discontinue their treatment or withdraw from participation in the study at any point in time. A number of seven patients dropped out during the study.

### Statistical analyses

To examine changes in general symptoms, schemas, and schema modes over time, we conducted repeated measures analysis of variance (mixed method ANCOVA) for each variable across two trial periods: pre- and post-treatment. Reliability was assessed using Cronbach’s alpha. This statistical measure reflects the internal consistency of the utilized scales. To assess the effect size, we calculated (generalized) Eta Squared for the Young Schema Questionnaire (YSQ), Schema Mode Inventory (SMI), and Symptom Questionnaire-48 (SQ-48). Effect sizes ranging from 0.01 to 0.05 are considered small, those between 0.06 and 0.13 are considered moderate, and effect sizes above 0.14 are considered large (Field, 2013). To address potential confounding factors, we controlled for DSM classification in the analyses by including it as a between-subject effect factor (covariate) and performed *post hoc* tests.” For the second aim of this study, we decided not to perform drop-out analyses when the attrition rate is below 10%. All statistical tests were two-tailed with a significance level of 5%. The statistical software JASP (Department of Psychological Methods, University of Amsterdam, Amsterdam, Netherlands) for Windows was employed for data analysis.

## Results

### Attrition

A number of 77 patients participated in the study. Of these, 25 percent were male. The age of the participants ranged between 20 and 66 years (*M* = 38, SD = 12.17). Approximately 21 percent had a PD (*N* = 15), 33 percent an anxiety disorder (*N* = 23) and the remaining 56 percent was diagnosed with a depressive disorder (*N* = 32). Seven participants withdrew from the study due to preference for individual therapy, or need to intensify therapy. Among the patients who did not complete therapy, two were diagnosed with a recurrent mood disorder; two with a recurrent anxiety disorder, and three with a PD. The participants who dropped out were excluded from the analyses and therefore the analyses were conducted solely on those who completed the treatment. The dropout rate in this study was 10% (see also Table 1).

**Table 1:** Sociodemographic data.

	Number of patients	Percentage
*Sex*		
Male	25	33
Female	52	67
*DSM5 diagnosis*		
Anxiety disorder	23	33
Mood disorder	32	56
Personality disorder	15	21
Obsessive compulsive	3	
Avoidant	3	
Otherwise specified	9	
Drop out	7	10
Injust indication	4	
Preference for individual therapy	3	

### Outcomes

All measures show a consistent pattern across all three diagnostic groups, with a tendency for reduction in scores throughout the treatment process. There were no significant differences between the DSM-5 classifications regarding changes in the aforementioned measures, except for the maladaptive modes where there was a significant interaction effect (see Table 2). *Post-hoc* tests revealed significant differences between patients with a mood disorder when compared to patients with either an anxiety disorder (*p* < 0.001) or PD (*p* < 0.001) at the second timepoint, as shown in Table 2. Patients with a mood disorder experienced more positive changes in maladaptive modes compared to patients with anxiety disorders and PDs. Effect sizes between pre-therapy and post-therapy scores, measured by the statistic eta^2^ were large for all measures, and these values are included in Table 2. Reliability scores for all measures were acceptable (see Table 3)

**Table 2:** Means, standard deviations, *F* statistics, and *p*-values of all questionnaire measures as a result of the repeated measures ANOVA.

Measure	Pre (SD)	Post (SD)	*F* (*p* value)	Eta^2^	Interaction effect
YSQ total			6.75 (<0.012)	0.21	0.55 (0.579)
Anxiety	2.95 (0.63)	2.43 (0.57)			
Depression	2.85 (0.67)	2.05 (0.50)			
PD	2.70 (0.63)	1.97 (0.35)			
SMI maladaptive			68.84 (<0.001)	0.28	3.18 (0.048)
Anxiety	2.86 (0.42)	2.35 (0.49)			
Depression	3.04 (0.77)	2.05 (0.53)			
PD	2.76 (0.49)	2.12 (0.32)			
SMI adaptive			67.29 (<0.001)	0.22	0.51 (0.60)
Anxiety	3.44 (0.52)	4.14 (0.61)			
Depression	3.55 (0.67)	4.32 (0.68)			
PD	3.74 (0.69)	4.30 (0.38)			
SQ-48 total			225.56 (<0.001)	0.42	0.83 (0.42)
Anxiety	70.26 (17.66)	41.83 (17.17)			
Depression	69.31 (16.67)	35.50 (16.26)			
PD	60.40 (18.84)	30.87 (17.99)			

**Table 3:** Reliability scores of the YSQ total score, SMI adaptive modes subscales, SMI maladaptive subscales and SQ048 total score subscale.

Measure	Reliability (Cronbach’s alpha)
*YSQ*	
Total score	0.77
*SMI*	
Adaptive modes	0.82
Maladaptive modes	0.70
*SQ-48*	
Total score	0.72

Regarding the second aim of this study, we found that approximately 10 percent of patients dropped out of therapy, indicating lower dropout rates than those reported in other studies using regular or short-term ST (Jacob and Arntz, 2013; Renner et al., 2013; Skewes et al., 2015).

## Discussion

The primary objective of the current study was to examine the feasibility of implementing a short-term, manualized form of ST in primary care settings and to assess changes in EMS, modes, and general psychological wellbeing among patients with either a PD and/or a therapy resistant depressive disorder and/or anxiety disorder. The reliability scores suggest a strong internal consistency of the utilized measurement instruments. This enhances the credibility of our findings and supports the validity of the results.

The results revealed a substantial effect size for the group between pre- and post-treatment across all measures. Patients exhibited a reduction in the total mean scores on the Young Schema Questionnaire (YSQ), indicating a decrease in negative core beliefs about oneself and interpersonal relationships. Additionally, both maladaptive and adaptive modes exhibited significant time effects, indicating that participants experienced changes in their cognitive and/or emotional patterns. This suggests that short-term schema therapy could be effective in addressing both dysfunctional and functional aspects of schema modes. Patients also reported improved overall psychological well-being. This positive trend indicates that participants experienced a meaningful enhancement in their mental health.

An important observation is the absence of significant differences in treatment outcomes between the three different DSM-5 diagnostic classifications, except for the maladaptive modes. Maladaptive modes exhibit notable resistance to change owing to their origins as early survival strategies, which play a pivotal role in shaping an individual’s sense of self. Consequently, the attributes of flexibility and openness to change, indicative of healthy adaptation, become significantly constrained (Young et al., 2003; Flanagan, 2010). Patients with a mood disorder experienced the most positive change in maladaptive modes, followed by patients with a personality disorder. Patients with an anxiety disorder experienced the least change in scores on maladaptive modes. Patients with a mood disorder seemed to experience more positive change in maladaptive modes compared to patients with anxiety disorders and PDs. The overall findings underscore the versatility of the approach in addressing a broad spectrum of psychological conditions.

The large effect sizes observed in all questionnaire measures further emphasize the clinical significance of the treatment outcomes. These effect sizes highlight the substantial changes that occurred between pre-therapy and post-therapy assessments, underscoring the relevance of the findings. Findings are in accordance with other studies on short-term ST for different patient groups, meaning that these effect sizes appear to be robust and characteristic of the intervention. In these studies, patients showed the same changes as in the current study, as well as changes in avoidant personality disorder symptom severity (van Vreeswijk et al., 2012; Renner et al., 2013; Skewes et al., 2015).

The attrition rates in this study (a dropout rate of 10 percent) are lower compared to other studies where they vary between 21 and 25% (Giesen-Bloo et al., 2006; Simpson H. B. et al., 2010; Simpson S. G. et al., 2010; van Vreeswijk et al., 2012), which is a promising observation for the implementation of Re-Focus in primary care. When compared to these studies, the main differences are the duration of therapy and the ability to meet the requirements of therapy in the study by Giesen-Bloo and colleagues, and the complexity of the problems with which patients registered for treatment, i.e., eating disorders, in the studies by Simpson et al. and the focus on cognitive techniques only in the study by Van Vreeswijk et al.

Strengths of the present study include the use of a comprehensive set of outcome measures, including assessments of Early Maladaptive Schemas (EMS), modes, and general psychological well-being. This comprehensive approach allows for a thorough evaluation of the intervention’s impact on various aspects of participants’ mental health. Lastly, the study’s findings have practical relevance for primary care settings, suggesting that short-term schema therapy can be successfully implemented for diverse patient groups in primary mental health care. This is important for clinicians and healthcare providers looking to offer effective interventions within the constraints of primary care, providing more continuity of care. Lastly, as a pilot study, naturalistic designs are clinically useful and have high ecological validity as the examined target population might constitute a direct reflection of the demographic cohort treated in practice (Lincoln and Guba, 1985; van Vreeswijk et al., 2012).

### Limitations

The present study has several limitations. Firstly, it lacked a randomized design, resulting in the absence of a comparative control group. Consequently, it is impossible to attribute the observed effects solely to the therapy. Secondly, a follow-up period was not incorporated, preventing the assessment of the long-term sustainability of the observed effects. Due to the study’s design, results may also be influenced by other factors, such as group effects versus individual therapy, the absence of a waiting list, and the passage of time. Future research should aim to control for these factors to attribute effects specifically to the treatment.

### Implications

The early findings of our pilot study suggest that patients with heterogeneous psychiatric conditions in primary care, who continue to experience symptoms or relapses after receiving standard care such as Cognitive Behavioral Therapy (CBT), may benefit from short-term group Schema Therapy (ST), specifically Re-Focus. These results imply that individuals with these disorders might find applicable treatment in primary care settings using a shorter and standardized version of ST. If future research confirms the viability of this treatment approach, it could facilitate more accurate guidance of patients toward the most appropriate form of therapy, resulting in reduced waiting times and lower costs for mental healthcare. This study also contributes to a growing body of literature that suggests that short-term ST shows promise as an intervention for patients with personality pathology and other disorders.

In conclusion, the results of this study demonstrate promising outcomes for short-term schema therapy. The relatively low attrition rates and significant improvements in various outcome measures suggest that this approach could be effective in enhancing psychological well-being and addressing schema-related issues. Furthermore, the treatment appears to be applicable across different diagnostic categories, emphasizing its potential as a versatile therapeutic approach. However, further research is needed to explore the reasons behind attrition and to confirm the long-term sustainability of these positive treatment effects. Furthermore, additional qualitative material is available and will be presented in a further study on the qualitative aspects of the Re-Focus.

## Data availability statement

The original contributions presented in the study are included in the article/Supplementary material, further inquiries can be directed to the corresponding author.

## Ethics statement

The studies involving humans were approved by the Commission Scientific Research and Participation (CSRP) of Vincent van Gogh. The studies were conducted in accordance with the local legislation and institutional requirements. The participants provided their written informed consent to participate in this study.

## Author contributions

IK: Writing – original draft. HH: Supervision, Writing – review & editing.

## References

[ref1008] American Psychiatric Association D. S. M. T. F. American Psychiatric Association. (2013). Diagnostic and statistical manual of mental disorders: DSM-5 (Vol. 5, No. 5). Washington, DC: American psychiatric association.

[ref1] Arntz A. Jacob G. A. Lee C. W. Brand-de Wilde O. M. Fassbinder E. Harper R. P.. (2022). Effectiveness of predominantly group schema therapy and combined individual and group schema therapy for borderline personality disorder: a randomized clinical trial. JAMA Psychiatry 79, 287–299. doi: 10.1001/jamapsychiatry.2022.0010, PMID: 35234828 PMC8892362

[ref5] Beckwith H. Moran P. F. Reilly J. (2014). Personality disorder prevalence in psychiatric outpatients: a systematic literature review. Personal. Ment. Health 8, 91–101. doi: 10.1002/pmh.1252, PMID: 24431304

[ref7] Carlier I. V. Kovacs V. van Noorden M. S. van der Feltz-Cornelis C. Mooij N. Schulte-van Maaren Y. W.. (2017). Evaluating the responsiveness to therapeutic change with routine outcome monitoring: a comparison of the symptom Questionnaire-48 (SQ-48) with the brief symptom inventory (BSI) and the outcome Questionnaire-45 (OQ-45). Clin. Psychol. Psychother. 24, 61–71. doi: 10.1002/cpp.1978, PMID: 26450457

[ref8] Carlier I. Schulte-Van Maaren Y. Wardenaar K. Giltay E. Van Noorden M. Vergeer P.. (2017). Development and validation of the 48-item symptom questionnaire (SQ-48) in patients with depressive, anxiety and somatoform disorders. Psychiatry Res. 200, 904–910. doi: 10.1016/j.psychres.2012.07.035, PMID: 22884307

[ref9] Carter J. D. McIntosh V. V. Jordan J. Porter R. J. Frampton C. M. Joyce P. R. (2013). Psychotherapy for depression: a randomized clinical trial comparing schema therapy and cognitive behavior therapy. J. Affect. Disord. 151, 500–505. doi: 10.1016/j.jad.2013.06.034, PMID: 23870427

[ref10] de Ruiter G. van Greuningen M. Luijk R. (2017). Inzicht in de geestelijke gezondheidszorg. Zorgthermometer GGZ, Vektis.

[ref1003] Durham R. C. Guthrie M. Morton R. V. Reid D. A. Treliving L. R. Fowler D. (2003). Tayside–Fife clinical trial of cognitive–behavioural therapy for medication-resistant psychotic symptoms: results to 3-month follow-up. British J. Psychiatry. 182, 303–311. 10.1192/bjp.182.4.303 12668405

[ref1004] Farrell J. M. Shaw I. A. Shaw I. (2012). Group schema therapy for borderline personality disorder: A step-by-step treatment manual with patient workbook. John Wiley & Sons.

[ref11] Field A. (2013). Discovering statistics with IBM SPSS statistics. Newbury Park, CA: Sage.

[ref12] Flanagan C. M. (2010). The case for needs in psychotherapy. J. Psychother. Integr. 20, 1–36. doi: 10.1037/a0018815

[ref1007] Fournier J. C. DeRubeis R. J. Shelton R. C. Hollon S. D. Amsterdam J. D. Gallop R. (2009). Prediction of response to medication and cognitive therapy in the treatment of moderate to severe depression. J. Consult. Clinical Psychology. 77:775. 10.1037/a0015401 PMC2810269 19634969

[ref13] Giesen-Bloo J. Van Dyck R. Spinhoven P. Van Tilburg W. Dirksen C. (2006). Outpatient psychotherapy for borderline personality disorder: randomized trial of schema-focused vs transference-focused psychotherapy. Arch. Gen. Psychiatry 63, 649–658. doi: 10.1001/archpsyc.63.6.649, PMID: 16754838

[ref14] Hawke L. D. Provencher M. D. (2011). Schema theory and schema therapy in mood and anxiety disorders: a review. J. Cogn. Psychother. 25, 257–276. doi: 10.1891/0889-8391.25.4.257 32759106

[ref16] Jacob G. A. Arntz A. (2013). Schema therapy for personality disorders—a review. Int. J. Cogn. Ther. 6, 171–185. doi: 10.1521/ijct.2013.6.2.171

[ref1002] Lincoln Y. S. Guba E. G. (1985). Naturalistic inquiry. sage.

[ref20] Lobbestael J. van Vreeswijk M. Spinhoven P. Schouten E. Arntz A. (2010). Reliability and validity of the short Schema mode inventory (SMI). Behav. Cogn. Psychother. 38, 437–458. doi: 10.1017/S1352465810000226, PMID: 20487590

[ref21] Loerinc A. G. Meuret A. E. Twohig M. P. Rosenfield D. Bluett E. J. Craske M. G. (2015). Response rates for CBTfor anxiety disorders: need for standardized criteria. Clin. Psychol. Rev. 42, 72–82. doi: 10.1016/j.cpr.2015.08.004 26319194

[ref22] Magnée T. Verhaak N. P. Boxem N. R. Onderhoud D. B. C. (2014). Verschuivingen van de tweedelijns geestelijke gezondheidszorg naar de eerstelijn en gevolgen daarvan voor de benodigde beroepsbeoefenaren: 2009–2012. Available at: http://www.nivel.nlnivel@nivel.nl

[ref23] Masley S. Gillanders D. Simpson S. Taylor M. (2011). A systematic review of the evidence base for schema therapy. Cogn. Behav. Ther. 41, 185–202. doi: 10.1080/16506073.2011.614274, PMID: 22074317

[ref25] Oei T. P. Baranoff J. (2007). Young Schema questionnaire: review of psychometric and measurement issues. Aust. J. Psychol. 59, 78–86. doi: 10.1080/00049530601148397

[ref27] Renner F. van Goor M. Huibers M. Arntz A. Butz B. Bernstein D. (2013). Short-term group schema cognitive-behavioral therapy for young adults with personality disorders and personality disorder features: associations with changes in symptomatic distress, schemas, schema modes and coping styles. Behav. Res. Ther. 51, 487–492. doi: 10.1016/j.brat.2013.05.011, PMID: 23778056

[ref28] Reubsaet R. J. (2018). Schematherapie: werken met fases in de klinische praktijk. Houten: Bohn Stafleu van Loghum.Diagnostiek van schema’s volgens het model van Young. Psychopraktijk 2, 29–30.

[ref29] Roca M. Gili M. Garcia-Garcia M. Salva J. Vives M. Campayo J. G.. (2009). Prevalence and comorbidity of common mental disorders in primary care. J. Affect. Disord. 119, 52–58. doi: 10.1016/j.jad.2009.03.014 19361865

[ref30] Rommelse N. Vreeswijk M. Van Broersen J. (2017). Kortdurende schematherapie: Handleiding kortdurende schematherapie: Voor groepstherapie en individuele therapie Houten: Bohn Stafleu van Loghum. ISBN 978-90-368-1546-8, doi: 10.1007/978-90-368-1547-5,

[ref31] Schene A. (2007). Preventie en de multidisciplinaire GGZ-richtlijnen. TSG 85, 63–64. doi: 10.1007/BF03078605

[ref32] Simpson S. G. Morrow E. van Vreeswijk M. F. Reid C. (2010). Group Schema therapy for eating disorders: a pilot study. Front. Psychol. 1:182. doi: 10.3389/fpsyg.2010.00182, PMID: 21833243 PMC3153792

[ref33] Simpson H. B. Neria Y. Lewis-Fernandez R. Schneier F.. (2010). Anxiety disorders – Theory, research and clinical perspectives. 1st Cambridge: Cambridge University Press.

[ref34] Skewes S. A. Samson R. A. Simpson S. G. van Vreeswijk M. (2015). Short-term group schema therapy for mixed personality disorders: a pilot study. Front. Psychol. 5:1592. doi: 10.3389/fpsyg.2014.01592 25657631 PMC4302795

[ref35] Springer K. S. Levy H. C. Tolin D. F. (2018). Remission in CBT for adult anxiety disorders: a meta-analysis. Clin. Psychol. 61, 1–8. doi: 10.1016/j.cpr.2018.03.002 29576326

[ref36] van Vreeswijk M. F. Broersen J. (2006). Schemagerichte Therapie in Groepen: Handleiding Voor Therapeuten. Houten: Bohn Stafleu van Loghum.

[ref37] van Vreeswijk M. F. Broersen J. (2013). Kortdurende Schemagroeps-Therapie: Cognitief Gedragstherapeutische Technieken Deel Handleiding, Rev. Edn. Houten: Bohn Stafleu van Loghum.

[ref38] Van Vreeswijk M. Broersen J. (2017). Handleiding kortdurende schematherapie: Voor groepstherapie en individuele therapie. Houten: Bohn Stafleu van Loghum, Springer.

[ref39] van Vreeswijk M. F. Spinhoven P. Eurelings-Bontekoe E. H. M. Broersen J. (2012). Changes in symptom severity, schemas and modes in heterogeneous psychiatric patientgroups following shortterm schema cognitive-behavioural group therapy: a naturalistic pre-posttreatment design in an outpatient clinic. Clin. Psychol. Psychother. 21, 29–38. doi: 10.1002/cpp.1813, PMID: 22933391

[ref41] Young J. E. (1990).Cognitive therapy for personality disorders: aschema-focused approach, Sarasota: Professional Resource Exchange.

[ref42] Young J. E. Klosko J. S. Weishaar M. E. (2003). Schema therapy: a practitioner’s guide. New York, NY: Guilford Press.

